# Effect of Combination of Point-of-Care C-Reactive Protein Testing and General Practitioner Education and Long-Term Effect of Education on Reducing Antibiotic Prescribing for Children Presenting with Acute Infections in General Practice in Latvia: A Randomized Controlled Intervention Study

**DOI:** 10.3390/antibiotics13090867

**Published:** 2024-09-10

**Authors:** Zane Likopa, Anda Kivite-Urtane, Ieva Strele, Jana Pavare

**Affiliations:** 1 Children’s Clinical University Hospital, Vienibas Gatve 45, LV-1004 Riga, Latvia; jana.pavare@rsu.lv; 2 Department of Paediatrics, Riga Stradins University, Vienības Gatve 45, LV-1007 Riga, Latvia; 3 Department of Public Health and Epidemiology, Institute of Public Health, Riga Stradins University, Kronvalda Boulevard 9, LV-1010 Riga, Latvia; anda.kivite-urtane@rsu.lv; 4 Institute of Occupational Safety and Environmental Health, Riga Stradins University, Dzirciema 16, LV-1007 Riga, Latvia; ieva.strele@rsu.lv

**Keywords:** acute infections, children, antibiotic prescription, general practice, point-of-care testing, education

## Abstract

Background: Antibiotics are often overprescribed in children in general practice. We investigated whether the availability of C-reactive protein point-of-care testing (CRP POCT) in daily practice and general practitioner (GP) education reduces antibiotic prescribing for children with acute infections and whether GP education has a long-term effect on antibiotic prescribing. Methods: This was a randomized controlled intervention study with randomization at the GP practice level. Eligible patients were children aged 1 month to 17 years presenting to general practice with an acute infection. Interventions: In the first study period, one GP group received combined interventions (CRP POCT was provided for daily use in combination with a live educational session), while the second GP group continued usual care. During the second study period, the GP groups were switched. During this period, the long-term education effect was evaluated in the GP group, which had previously received both interventions: the CRP POCT was no longer available in their practices in accordance with the study protocol, but education could have a lasting effect. Primary outcome: Antibiotic prescribing at index consultation. Results: GP with combined intervention enrolled 1784 patients, GP with usual care enrolled 886 patients, and GP with long-term education effect enrolled 647 patients. Most of the patients had upper (76.8%) and lower (18.8%) respiratory infections. In total, 29.3% of the study patients received antibiotic prescriptions. Adjusted binary logistic regression analysis showed no differences for the primary outcome between GPs with usual care and GPs with combined intervention (aOR 0.89 (0.74–1.07), *p* = 0.20), but significantly lower antibiotic prescribing was observed for GPs with long-term education in comparison with GPs with usual care (aOR 0.75 (0.59–0.96), *p* = 0.02); however, after multilevel analyses, any differences in the antibiotic prescription between intervention groups became non-significant. GPs widely used CRP POCT when it was available in practice (for 69.1% of patients in the combined intervention group), but rarely measured CRP in the laboratory in the usual care group (8.8% (n = 78)) or long-term education group (14.8% (n = 98)). The majority of the tested patients had low CRP levels (below 20 mg/L); despite this, up to 35.4% of them received antibiotic prescriptions. Conclusions: Our results show that the availability of CRP POCT and educational training for GPs together did not reduce antibiotic prescribing, and one-time education did not have a long-term effect on antibiotic prescribing.

## 1. Introduction

Overuse of antibiotics with increasing bacterial resistance is considered a major threat to human health around the world [1]. The European Academy of Paediatrics stated that large variations in antibiotic prescriptions and overprescribing still exist across Europe and that initiatives to decrease antibiotic use are necessary [2]. General practice accounts for around 80% of all antibiotic prescriptions [3]. Antibiotics are often prescribed for children with acute diseases, such as respiratory and ear infections, as well as urinary, skin, and soft tissue infections, which are often managed by GPs [4]. Meanwhile, self-limiting viral infections are predominant in children and do not require specific treatment [3,5].

Unnecessary prescribing is a highly complex phenomenon. The main drivers of overprescription are diagnostic and prognostic uncertainty, limited visit time, heavy workloads, and GP unfamiliarity with recent guidelines; there can also be expectations and pressure from patients and parents to be given antibacterial treatment [6,7,8,9]. These factors highlight the main targets for interventions to reduce antibiotic prescribing, and previously, many of them have been carried out with varying results in both children and adults [10]. Multifaceted interventions tend to be the most successful [11,12].

We aimed to assess the effect of the combination of two interventions that could reduce diagnostic uncertainty on antibiotic prescription: access to the use of C-reactive protein point-of-care testing (CRP POCT) and GPs education training. In addition, the study model allowed us to evaluate the long-term effect of the received education.

Prescribing decisions are often made by a GP using traditional but non-evidence-based clinical signs, such as sputum production, fever, chest signs, and feeling unwell [3]; however, these are rarely supported by laboratory tests. Testing that could help clinicians diagnose infections accurately and rapidly and determine which patients will benefit from antibiotics is crucial [1]. By providing immediate results, POCT may support clinical decision-making and optimize antibiotic prescribing in general practice. CRP is a marker of an acute inflammatory response and, in the last few years, has been widely introduced as a POCT that is especially appropriate for general practice [13]. CRP POCT has reduced antibiotic prescribing for adults [14] and has been adopted by the National Institute for Health and Clinical Excellence (NICE) for adults with lower respiratory infections [15]; however, for children, it is used more as a by-product of the test being made available for adult patients because data are presently incomplete and controversial and require further analyses [13,15,16]. The results of a previous study in the adult population suggest that an intervention combining CRP testing and education had the largest effect on prescribing habits [17]; however, data on the age of children are scarce [5].

## 2. Results

### 2.1. General Practitioner and Patient Flow

**Figure 1:**
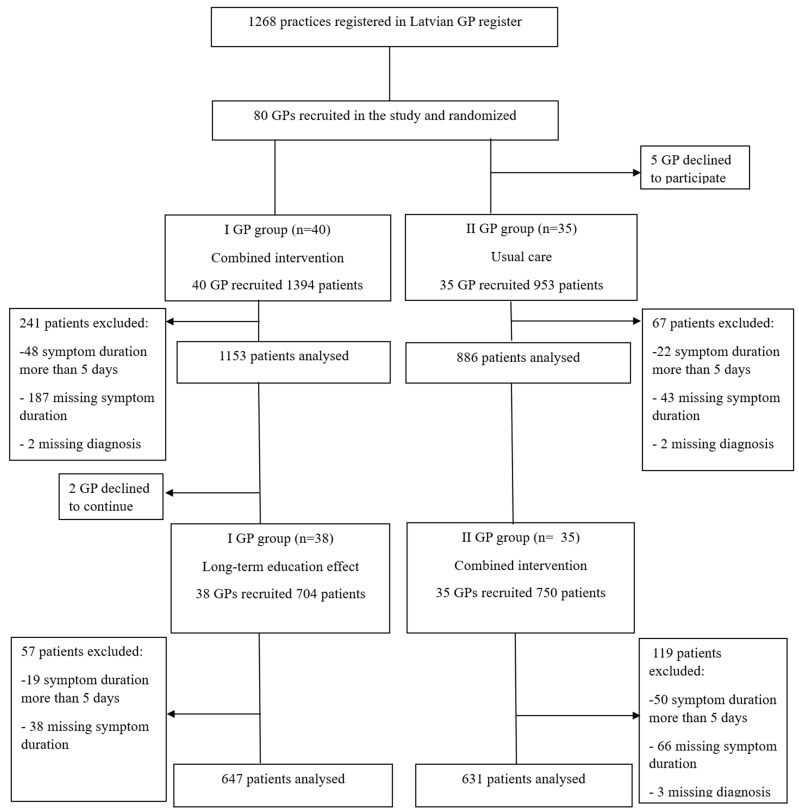
Flowchart of the study recruitment process. GP: general practitioner.

Eighty GPs started the study, of whom 40 were assigned to the combined intervention and 40 to usual care (Figure 1). Five GPs from the usual care group declined to participate after randomization and two GPs refused to participate in the second period of the study. In total, 3801 patients were enrolled in the study. Of these, 484 patients were excluded due to noncompliance with the study inclusion criteria or incomplete data regarding symptom duration or diagnoses. Overall, 1784 patients were recruited by GPs allocated to combined intervention, 886 by GPs allocated to usual care, and 647 by GPs allocated to long-term education effect.

### 2.2. General Practitioner Baseline Characteristics

The median age of the GPs was 53.0 years (interquartile range (IQR) 46.0–61.0), with work experience ranging from 1 year to 52 years. Only two GPs were male. The number of pediatric patients varied considerably between practices, ranging from 48 to 1843 children. The study selected practices from all five regions of Latvia, including 24 practices from rural regions, 18 from regional cities, and 33 from the capital city.

### 2.3. Patient Baseline Characteristics

The median age of the patients was 4.0 (IQR 2.0–8.0) years, and most patients were small children in the age group of 0–4 years (n = 1686, 51.3%). Both sexes were equally presented (50.7% male, 49.3% female). The median duration of symptoms was 3 days (IQR 2.0–4.0). Only 8.2% of the study population (n = 271) reported any underlying condition, the most frequent being bronchial asthma (n = 223, 85.1%). Most patients visited a GP because of respiratory symptoms, specifically 2546 (76.8%) with an upper respiratory tract infection and 625 (18.8%) with a lower respiratory tract infection. Fifty-three patients were referred to the hospital after the visit, and all other patients were treated in general practice.

The baseline characteristics of the patients in the three analyzed groups are detailed in Table 1. It can be seen that the studied groups differ statistically significantly according to all selected characteristics except for sex. Thus, the long-term education effect group has a significantly higher proportion of young children, of those with urinary tract or soft tissue infections, and of responders who have been referred to the hospital, while a lower proportion of children with chronic conditions and of those fully vaccinated.

### 2.4. Effects of Interventions on Antibiotic Prescribing

**Figure 2:**
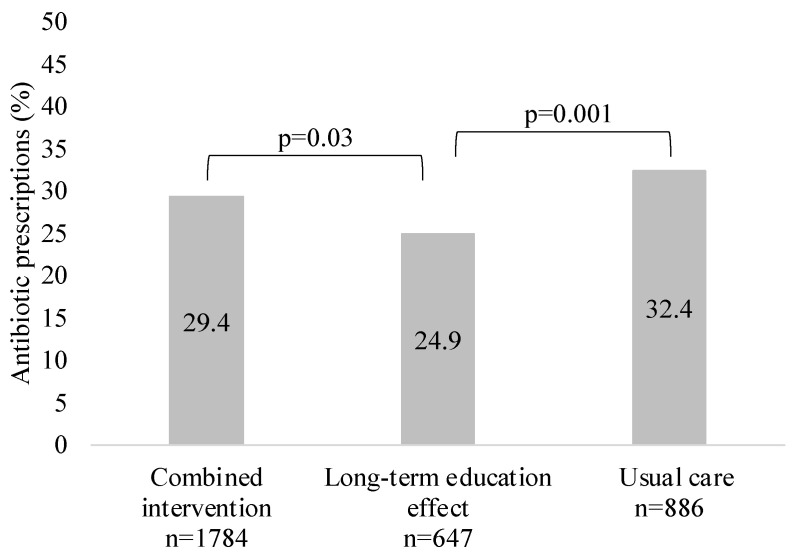
Proportion of patients (%) treated with antibiotics in each study group.

Overall, 29.3% of the study patients (n = 972) received antibacterial treatment (immediate or delayed prescription). The lowest prescribing was observed in the GP group, with a long-term education effect. The proportion of patients treated with antibiotics in each study group is shown in Figure 2.

Further logistic regression analyses were performed for possible covariates (Table 2), and we observed that antibiotic prescribing was significantly associated with girls’ sex (OR vs. boys 1.17, *p* = 0.045), longer duration of symptoms (OR was 1.88, *p* = 0.001, 2.24, *p* < 0.001 and 1.93, *p* = 0.002 for days 3, 4 and 5, respectively, compared to 1 day), middle-age and 61+ GP (OR for GP aged 41–50 years vs. 30–40 years was 2.05, *p* < 0.001; for GP aged 61+ vs. 30–40 years—1.38, *p* = 0.006)), and fewer pediatric patients in practice (aOR was 1.39, *p* = 0.007 and 1.50, *p* < 0.001 for <500 and 501–1000 patients, respectively, compared to >1001 patients). However, after adjustment, the association remained statistically significant for younger children (aOR for children aged 10–14 years vs. 0–4 years was 0.74, *p* = 0.03), longer duration of symptoms (aOR for day 4 vs. day 1 was 1.66, *p* = 0.02), and fewer pediatric patients (aOR 1.76, *p* = 0.04 for practices with 501–1000 patients vs. 1000+). After adjusting for covariates before multilevel modeling, education intervention in the long term maintained a significant effect on antibiotic prescribing reduction in comparison with usual care (aOR 0.75 (0.59–0.96), *p* < 0.02), but CRP POCT and education intervention together did not influence prescribing (aOR 0.89 (0.74–1.07), *p* = 0.20). However, when GP practices were included in the model as the second-level variable indicating clusters, any differences in antibiotic prescription between the intervention groups became non-significant. The variance partition coefficient (VPC) was 19.4%, meaning that almost 20% of the total variation in antibiotic prescribing was attributable to differences between GP practices (*p* < 0.001).

### 2.5. CRP POCT Results and Their Association with Antibiotic Prescribing for Patients Visiting GPs in Different Study Groups

In total, 69.1% (n = 1233) of patients managed by GPs in the combined intervention group were tested for CRP; meanwhile, CRP was rarely measured in the laboratory in the usual care group (8.8% (n = 78)) or long-term education group (14.8% (n = 98)), and the difference was statistically significant (*p* < 0.001).

**Figure 3:**
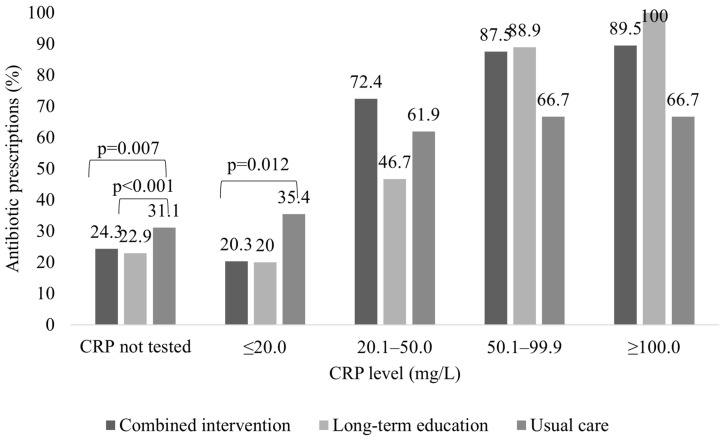
Comparison of antibiotic prescription for patients visiting GPs in the control group, combined intervention group, and long-term education group according to CRP level.

Antibiotics were more often prescribed to patients without CRP testing in the usual care group than in the combined intervention (31.1% vs. 24.3%, *p* = 0.007) or long-term education group (31.1% vs. 22.9%, *p* < 0.001). Similarly, the combined intervention group prescribed significantly fewer antibiotics for patients with CRP below 20 mg/L than the usual care group (20.3% vs. 35.4%, *p* = 0.012). Patients with CRP levels above 20 mg/L were more frequently treated with antibiotics in all study groups, and no significant differences were observed between the groups for these patients. (Figure 3).

Patients who were not prescribed antibiotics despite a CRP value of > 100 mg/L were referred to secondary care.

## 3. Materials and Methods

The study was carried out in general practice settings in Latvia between November 2019 and April 2021. This was a randomized controlled intervention study with randomization at the GP practice level.

For the purposes of this study, we had access to 40 CRP POCT devices, enabling 80 GPs to be recruited. The participating GP were recruited using two approaches. First, from the country’s 1268 GP, by means of an Excel random number generator, we selected 160 GP (the expected response rate was 50%) across different geographically located practices (urban and rural areas) and sent invitations to participate in the study via both email and paper-based letter forms. Unfortunately, the response rate was lower than expected and only 38 participants were recruited using this approach. Secondly, we directly addressed GP at a meeting of the Latvian General Practitioners Association and consequently achieved the requisite number of 80 participants. The 80 GPs who consented to the study were stratified according to practice location, and each stratum was divided into two groups of 40 GPs using random numbers generated by the MS Excel Random Number function. Detailed information on the GP recruitment process can be found elsewhere [18].

We chose a study design with 2 periods, where each of the participating GPs had the opportunity to receive both interventions for one period, thus providing additional motivation to participate and allowing more patients to be included in the study.

**Figure 4:**
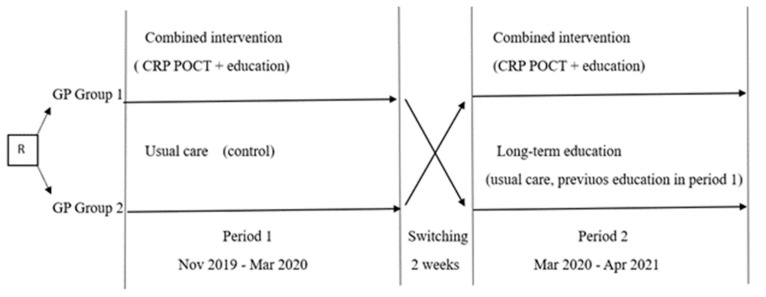
Study overview. R: randomization. GP: general practitioner. CRP POCT—C-reactive protein point-of-care test.

In the first period, the GPs in Group 1 received two interventions (CRP POCT and a live educational session), and Group 2 continued usual care as a control (Figure 4). Recruitment during the first period took place between November 2019 and March 2020. The results of the first period of the study are published [19].

Within two weeks after the end of the first period, participating GP groups were switched for the second period: GPs with usual care in the first period received combined interventions for the second period, but GPs who previously received combined interventions continued usual care without access to CRP POCT. However, as this group had received education intervention in the first study period, we analyzed the data of this group separately, evaluating the long-term effect of education.

As a result, data from the two GP groups according to the presence of intervention were combined for analysis as follows:

- GP with usual care (GP Group 2 in the 1st period)
- GP with combined intervention (GP Group 1 in the 1st period plus GP Group 2 in the 2nd period)
- GP with long-term education effect (GP Group 1 in the 2nd period)

Recruitment of patients in the second period was significantly extended due to a reduced number of acute illness episodes in GP practices in the spring of 2020 as a consequence of SARS-CoV-2 virus (COVID-19) epidemiological safety measures, and took place between March 2020 and April 2021.

### 3.1. Interventions

We evaluated the effect of a combination of two interventions: a live educational session and a CRP POCT.

The four-hour live educational session included the following:

- principles of antibiotic resistance and safer prescribing of antibiotics, narrow—spectrum antibiotic prescribing strategy, criteria for antibiotic prescribing, principles of optimal antimicrobial treatment duration (conducted by Latvian Health Ministry’s chief infectologist)
- new recommendations for the management of upper and lower respiratory infections (patient evaluation, diagnostics, treatment) introduced in 2019 in Latvia (conducted by a Children University Hospital pulmonologist, one of the authors of recommendations)
- new recommendations for the management of fever (patient evaluation, precautionary levels (risk levels), diagnostics, laboratory testing, and treatment) were introduced in 2019 in Latvia (conducted by a pediatrician at Children University Hospital, one of the authors of recommendations).

GPs also received educational materials in video and printed formats after the session.

GPs were administered using the Orion Diagnostica QuikRead go CRP POCT system for the quantitative determination of CRP in blood, with a sample volume of 20 µL obtained via a fingerprick. This system has a measurement range of 5–200 mg/L, and the results are available within 2 min. As CRP cutoff levels for children in general practice are currently undetermined [20], the GPs did not receive any guidelines for the interpretation of results. Each GP was individually tutored by the diagnostic test company on how to perform the CRP test during a face-to-face meeting, and ongoing support from the company was available to the GP during the intervention period. GPs ordered a CRP test only if they believed that the result would help them make a more informed decision on antibiotic necessity after a clinical assessment.

### 3.2. Data Collection

All participating GPs were asked to recruit child patients (1 month up to 17 years old) who visited them in face-to-face visits with clinical signs of an acute infection. We included patients who had symptoms lasting less than 5 days, who were not in the recovery stage, and who had not started antibiotics at the time of the visit. Recruitment rates across GPs were monitored during the study period through regular telephone calls and visits. Patient demographics, diagnosis, investigations, and decisions on antibacterial treatment were registered for all included patients.

The primary outcome was antibiotic prescribing during the index consultation. Furthermore, patient- and GP-related predictors of antibiotic prescribing and CRP levels were also analyzed.

The study was approved by the Research Ethics Committee of Riga Stradins University, approval no. 6-3/5/21 (received: 30 May 2019).

### 3.3. Sample Size

We presumed that the frequency of antibiotic prescribing in the intervention group compared to the control group was 34% and 42%, respectively, as per Martínez-González et al. [21]. Thus, according to Fleiss et al. [22], with 80% power and α level of 5%, our study required 571 patients in each study group.

### 3.4. Statistical Analyses

Descriptive statistics, such as medians (with interquartile range) for continuous variables (as the data did not meet the conditions of normal distribution, checked with Kolmogorov-Smirnov Test) and proportions for categorical variables, were calculated. The Chi-square Test or Fisher’s Exact Test was used to determine the statistical significance of differences in the proportions of dependent variables between subgroups of independent variables, whereas for continuous variables—the Kruskal-Wallis Test and Mann-Whitney Test were used. Univariate and multivariate binary logistic regression were used to identify factors associated with antibiotic prescription. To account for the potential clustering of observations in GP practices, the associations were also tested using a random intercept logistic regression model where 75 GP practices were included as second-level units. The variance partition coefficient (VPC) was calculated using the latent variable method [23].

Results were considered statistically significant at *p* < 0.05. Data processing was performed using IBM SPSS Statistics (Statistical Package for the Social Sciences, Version 26.0).

## 4. Discussion

### 4.1. Main Findings

The combination of GP access to CRP POCT and face-to-face education intervention in comparison to usual care did not result in a significant reduction in antibiotic prescribing for children with acute illnesses. Although we observed a slight reduction in antibiotic prescribing in the long-term education group, the significance disappeared after multilevel analyses.

Overall, we observed a low antibiotic prescribing level for children with acute illnesses (29.3%). Multivariate analysis revealed that several patient-related factors (younger children, girls, longer duration of symptoms) and GP-related factors (middle-aged GPs, fewer pediatric patients in practice) were significantly associated with antibiotic prescription. The effect remained for younger children, longer duration of symptoms, and fewer pediatric patients in practice after multilevel analyses.

GPs often considered CRP POCT once it was available in practice (69.1%) but rarely used CRP in the control group and long-term education group when CRP point-of-care testing was not available. Meanwhile, the long-term education group used laboratory CRP testing more often than the usual care group (14.8% v 8.8%, *p* < 0.001).

Despite the low CRP level measured (<20 mg/L), GP often preferred to prescribe antibiotics, although serious bacterial infections would be unlikely for these patients. However, we found a significant decrease in antibiotic prescribing for patients without CRP testing in the combined intervention group and long-term education group and for patients with low CRP levels (<20 mg/L) in the combined intervention group.

### 4.2. Strengths and Limitations

Practices were recruited from different Latvian regions, including urban and rural practices, and stratified according to practice location. Additionally, large variations were observed in the number of pediatric patients registered in the practices, thus providing representative data of Latvian general practice. As CRP POCT is not incorporated into Latvian general practice, it provides an opportunity to better establish the effect of this intervention. In addition, this was the first time GPs were introduced to new clinical pathways and guidelines.

We achieved the targeted patient number and included the total childhood population, except up to 1 month of age, as this group of patients with acute illnesses is mostly recommended to be sent to the hospital.

One limitation is that not all consecutive patients visiting GPs were included in the study due to time constraints during the GPs’ consultation hours.

A further limitation is that we analyzed only antibiotic prescriptions in the index consultation and that the clinical outcomes of the recruited patients or later antibiotic prescriptions were not considered. In addition, we did not have clinical data on the severity of episodes for the included patients, but only the diagnoses established by GPs.

We observed a low overall antibiotic prescription level; one reason for this could be that GPs might have altered their antibiotic prescribing habits due to participation in the study. However, the focus of GPs was on the opportunity to trial the CRP POCT and evaluate its usefulness in daily practice rather than on the outcome measures of the study; therefore, it is unlikely that meaningful changes in routine practice occurred. Furthermore, it was not possible for us to perform a completely randomized selection of GPs for the study. GPs participated voluntarily, which could indicate their above-average interest in accepting new knowledge and improving their daily practices.

### 4.3. Comparison with Existing Literature

This study focused on the impact of the combination of CRP POCT and GP education intervention. In addition, the study model allowed for the evaluation of the long-lasting effect of GP education. As far as we know, it is the first time the combination of these factors in general practice for child-age patients has been evaluated. Previously, the effects of education and CRP POCT have been evaluated in adolescents and adults, and antibiotic prescribing was reduced from 36% to 24% [24]. For children, CRP POCT, in combination with communication training, has resulted in a positive effect in reducing antibiotic prescribing for respiratory infections in general practice [25].

Although a trend of reduction in antibiotic prescriptions was observed, it was not possible to reach a statistically significant effect for the combination of CRP POCT and GP education intervention or long-term education. Despite this, the small effect should be seen in light of the overall low antibiotic prescribing rates (29.3% of study patients received antibiotic prescriptions). In line with previous studies, interventions are less effective in reducing antibiotic use in low-prescribing countries [26].

In recent years, CRP POCT has been widely introduced in general clinical practice and is a part of antibiotic stewardship programs [21]. Although the diagnostic value of the CRP POCT effect in general practice has been published in four systematic reviews, most of the studies included adults [21,27,28,29]. In the latest Cochrane review [27], only four studies were carried out exclusively among children, and the authors concluded that C-reactive protein likely reduces the number of children given an antibiotic prescription; however, the effect was primarily seen in low- and middle-income countries and could be lower in low baseline antibiotic prescribing countries. Previously, Martínez-González et al. in meta-analyses showed that CRP POCT overall significantly reduced immediate antibiotic prescribing at the index consultation for respiratory infections, but subgroup analysis by age groups showed no significantly lower antibiotic prescribing for children and highlighted that a more pronounced reduction of antibiotic prescribing for children was observed when CRP cutoff guidance was applied [21]. This is also consistent with a previous systematic review carried out by Verbakel et al. [28]. However, cutoff levels for children observed in general practice are not clearly defined [20] and may differ depending on age; therefore, we did not provide any guidelines for GP that could reduce the effect of this intervention in our study. Van den Bruel et al. used CRP levels < 20 mg/L to rule out serious bacterial infections and concluded that if antibiotics had been withheld for those children, it could reduce the overall antibiotic consumption by 15% [30]. Schot et al. also observed that 14% of children with a CRP level < 10 mg/L were prescribed antibiotics [31]. Do et al. observed a statistically significant effect on reducing antibiotic prescribing for both adults and children when recommendations were provided to withhold antibiotics when CRP was less than 10 mg/L for children younger than 5 years and less than 20 mg/L for others. We also observed that around 20% of children with CRP less than 20 mg/L still received antibiotic prescribing, with a higher prescribing level in the usual care group (35.4% vs. 20.3% in the combined intervention group and 20.0% in the long-term education group). This suggests the need for further research to promote recommendations on the value of POC CRP for children to guide antibiotic prescribing.

Physician knowledge, attitude, and behavior play important roles in mediating antimicrobial prescribing [32]; therefore, educational programs are established as an important part of antimicrobial stewardship to optimize antibiotic prescribing [7]. It is recommended that the fundamentals of antibiotic resistance and its relationship with antibiotic use, clinical evaluation, diagnosis and management of infection, and specific behavioral changes be focused on to improve antibiotic prescribing [33], and these principles were also used in our study. Previously, education interventions were more established in hospital settings than in general practice [32,34]. In our study, education did not have a significant effect on antibiotic reduction in the long-term, but multilevel analyses highlighted the importance of each GPs’s antibiotic prescribing habits. Previous studies have concluded that GPs’ have wide variations in diagnostic procedures and antibiotic prescribing, but these habits are quite consistent once established [35,36].

Possibly, it takes time for a GP to accept new knowledge and change prescribing behavior, as treatment decisions often rely on previous experience and familiarity with antibiotics [9]. We observed that GPs with work experience of more than 10 years and more durable treatment habits had higher prescribing rates, which is consistent with previous studies. [10]. Moreover, GPs with a longer practicing time have developed certain doctor-patient relationships and treatment expectations, which might influence GPs.

### 4.4. Implications for Research and Practice

Although we observed a low antibiotic prescribing rate, there is still room for improvement as acute illnesses are a very common complaint for children in general practice, and reasons for antibiotic usage and a part of prescriptions are still unnecessary. However, in low-prescribing countries, it is more difficult to achieve a reduction.

Diagnostic tests can play a role in reducing diagnostic uncertainty and in changing GP prescribing decisions; therefore, their incorporation into broader antibiotic stewardship programs should be considered [34]. However, the combination of POC CRP testing without guidelines and GP education was not effective in reducing antibiotic prescribing and should not be recommended. One possibility for the future is to determine safe cutoff levels for CRP in general practice according to patient age in order to enhance its effectiveness [27].

The findings of this study suggest that one-time education sessions did not have a lasting effect on antibiotic reduction. Additional research is needed to achieve the most effective and long-lasting education format for general practice. Previous studies have shown that a longer-lasting effect on prescribing habits could be achieved with personalized feedback and regular audits after education intervention.

## 5. Conclusions

The combination of CRP POCT and one-time GP education intervention does not have a significant effect on antibiotic prescribing when the generally low antibiotic prescription is observed.

Although GPs often consider CRP testing when POCT is available, a low CRP level does not always convince GPs to withhold antibiotics, which indicates that future research directions on the interpretation and accuracy of testing for child-age patients could improve its effectiveness.

## Figures and Tables

**Table 1:** Patients’ baseline characteristics managed by GP with combined intervention, usual care, and long-term education effect.

Variables	Combined Intervention (n = 1784)	Usual Care (n = 886)	Long-Term Education Effect (n = 647)	*p*
Age (years) Median (IQR) 0–4 years 5–9 years 10–14 years 15–17 years	5.0 (2.0–9.0) a 839 (47.6%) abc 569 (32.3%) a 249 (14.1%) b 107 (6.1%) c	5.0 (2.0–8.0) b 435 (49.7%) def 279 (31.8%) d 120 (13.7%) e 42 (4.8%) f	3.0 (2.0–6.0) ab 412 (63.8%) abcdef 159 (24.6%) ad 57 (8.8%) be 18 (2.8%) cf	**<0.001** **<0.001**
Sex Boys Girls	865 (48.8%) 907 (51.2%)	440 (50.0%) 440 (50.0%)	318 (49.5%) 325 (50.5%)	0.84
Duration of illness (days) Median (IQR)	3.0 (2.0–4.0) ab	3.0 (2.0–4.0) a	3.0 (2.0–4.0) b	**<0.001**
Chronic disease Yes No	126 (7.1%) a 1658 (92.9%) a	102 (11.5%) ab 784 (88.5%) ab	43 (6.6%) b 604 (93.4%) b	**<0.001**
Full vaccination Partial vaccination No vaccination	1623 (92.9%) a 105 (6.0%) 19 (1.1%) a	820 (95.0%) abc 40 (4.6%) b 3 (0.3%) ac	580 (91.3%) bc 44 (6.9%) b 11 (1.7%) c	**0.03**
Diagnoses Upper respiratory infection Lower respiratory infection Gastrointestinal infection Urinary tract infection Skin and soft tissue infection Bone and joint infection	1376 (77.1%) ab 337 (18.9%) cd 41 (2.3%) ef 20 (1.1%) ace 8 (0.4%) bdf 2 (0.1%)	675 (76.2%) gh 180 (20.3%) ij 19 (2.1%) k 10 (1.1%) gi 1 (0.1%) hjk 1 (0.1%)	495 (76.5%) abgh 108 (16.7%) cdij 13 (2.0%) efk 19 (2.9%) acegi 11 (1.7%) bdfhjk 1 (0.2%)	**<0.001**
Ambulatory patients Referred to hospital	1756 (98.4%) 28 (1.6%)	879 (99.2%) a 7 (0.8%) a	629 (97.2%) a 18 (2.8%) a	**0.009**

a, b, c, d, e, f, g, h, i, j, k—two-by-two tables earmarked where statistically significant differences have been found.

**Table 2:** Patient- and GP-related predictors of antibiotic prescribing.

Characteristics	Crude OR (95% CI)	*p*	Adjusted OR * (95% CI)	*p*
Patient-related factors				
Age (years) 15–17 10–14 5–9 0–4	1.21 (0.86–1.69) 0.80 (0.63–1.02) 0.93 (0.79–1.11) 1	0.27 0.07 0.44	1.16 (0.80–1.69) **0.74 (0.57–0.97)** 0.89 (0.73–1.07) 1	0.44 **0.03** 0.21
Sex Boys Girls	1 **1.17 (1.00–1.36)**	**0.045**	1 1.14 (0.97–1.34)	**0.13**
Duration of symptoms (days) 1 2 3 4 5	1 1.48 (1.00–2.18) **1.88 (1.28–2.76)** **2.24 (1.50–3.34)** **1.93 (1.26–2.95)**	0.05 **0.001** **<0.001** **0.002**	1 1.10 (0.72–1.67) 1.31 (0.86–1.98) **1.66 (1.08–2.57)** 1.50 (0.94–2.40)	0.66 0.21 **0.02** 0.09
**GP-related factors**				
Age (years) 30–40 41–50 51–60 61+	1 **2.05 (1.63–2.59)** 1.09 (0.87–1.37) **1.38 (1.09–1.73)**	**<0.001** 0.47 **0.006**	1 2.05 (0.96–4.40) 1.26 (0.64–2.50) 1.41 (0.68–2.90)	0.07 0.50 0.36
Sex Male Female	1 0.76 (0.47–1.23)	0.27	1 1.24 (0.27–5.59)	0.78
Work experience <5 years 6–10 years 11–20 years 21+ years	1 **0.40 (0.25–0.66)** 1.31 (0.97–1.77) **1.40 (1.08–1.80)**	**<0.001** 0.08 **0.01**	1 0.68 (0.19–2.39) 1.93 (0.73–5.10) 1.71 (0.78–3.77)	0.54 0.19 0.18
Location of practice Rural areas Regional cities Capital of Latvia	1.16 (0.98–1.38) 0.96 (0.79–1.18) 1	0.08 0.71	1.43 (0.79–2.58) 1.25 (0.69–2.28) 1	0.24 0.47
Number of pediatric patients in practice <500 501–1000 1001+	**1.39 (1.10–1.77)** **1.50 (1.28–1.76)** 1	**0.007** **<0.001**	1.31 (0.54–3.17) **1.76 (1.04–2.98)** 1	0.55 **0.04**
Expected time of laboratory results During working day Next working day 2 working days or longer	1 0.97 (0.84–1.13) 0.82 (0.42–1.58)	0.71 0.54	1 1.06 (0.66–1.72) 0.83 (0.12–5.74)	0.80 0.85
Study group Combined intervention Long-term education effect Usual care	0.87 (0.73–1.03) **0.69 (0.55–0.87)** 1	0.11 **0.001**	1.04 (0.81–1.34) 0.92 (0.65–1.31) 1	0.75 0.64

* Adjusted OR: adjusted odds ratio—adjusted for all independent variables in the table, except the age of GP due to collinearity with the duration of the career of GP (for the age of GP, variable adjustment was carried out for all the variables except the duration of career).

## Data Availability

The original contributions presented in the study are included in the article, and further inquiries can be directed to the corresponding author.
